# Dynamic functional connectivity of the amygdala-hippocampal complex is associated with cognitive impairment in adolescents with Internet gaming disorder

**DOI:** 10.3389/fpsyt.2025.1689119

**Published:** 2025-11-26

**Authors:** Tao Zhao, Shuyi Zhang, Qiyan Lv, Yange Li, Dingyi Li, Meijun Liu, Yan Lang

**Affiliations:** Department of Psychiatry, First Affiliated Hospital of Zhengzhou University, Zhengzhou, China

**Keywords:** internet gaming disorder, cognitive impairment, brain networks, dynamic functional network connectivity, adolescents

## Abstract

**Background:**

The amygdala-hippocampal complex (AHC) plays a central role in the neural mechanisms underlying Internet Gaming Disorder (IGD), particularly in emotional regulation, memory processing, and reward-related functions. However, the dynamic interactions between the AHC and large-scale brain networks, and their relationship with cognitive performance in IGD, remain poorly understood.

**Methods:**

A total of 123 adolescents (66 with IGD and 57 healthy controls) underwent resting-state functional magnetic resonance imaging (fMRI). Temporal fluctuations in AHC connectivity were assessed using dynamic functional network connectivity (dFNC) analysis. Correlation and mediation analyses were conducted to investigate the relationship between aberrant AHC-related dFNC and cognitive function.

**Results:**

Three distinct connectivity states were identified, each characterized by unique network configurations. In State 2, dFNC strength between the AHC and both the attentional network (ATN) and visual network (VN) was positively correlated with T scores of the MATRICS Consensus Cognitive Battery (MCCB). Further mediation analysis revealed that weakened dFNC between the AHC and VN regions, particularly the calcarine sulcus and cuneus, served as a mediator linking cognitive impairment to the internet addiction severity of IGD.

**Conclusion:**

These findings suggest that aberrant dynamic connectivity of the AHC, particularly its disrupted interaction with VN, may underlie the cognitive impairments in adolescents with IGD. This study provides novel insights into the neurobiological basis of behavioral addiction and highlights the importance of dynamic network analysis in elucidating its underlying pathology.

## 1 Introduction

Internet gaming disorder (IGD) is characterized by impaired control over gaming and persistent engagement in online games (1), often accompanied by physical and psychological symptoms, social dysfunction, and diminished academic or occupational performance (2). In the 11th edition of the International Classification of Diseases (ICD-11) (3), Gaming Disorder (GD) is formally recognized as a mental health condition resulting from addictive behaviors and is classified under “disorders due to addictive behaviors”. Recent epidemiological evidence points to a rising prevalence of IGD among adolescents (4, 5). Cognitive impairment is considered both a major risk factor for behavioral addiction and a core feature of IGD in youth (6, 7). Adolescents with IGD exhibit a wide range of cognitive deficits, which significantly disrupt daily functioning and academic performance, imposing substantial burdens on both affected individuals and their families (8). In light of these detrimental consequences, elucidating the neurobiological mechanisms underlying cognitive dysfunction in adolescent IGD is of critical importance (9).

The amygdala and hippocampus play pivotal roles in the pathophysiology of IGD. The amygdala is critically involved in emotional regulation (10), and individuals with IGD exhibit widespread structural and functional abnormalities within the amygdala-striatal circuitry (11). Notably, IGD patients with heightened impulsivity traits show reduced functional connectivity (FC) between the amygdala and the left inferior frontal gyrus (12), suggesting that alterations in amygdala gray matter density may underlie susceptibility to impulsivity in IGD (13). Furthermore, a radiomics-based classification model integrating features from the right caudate nucleus and the amygdala demonstrated superior IGD diagnostic accuracy (14), underscoring the amygdala’s potential as a neurobiological marker of the disorder. Individuals who engage in computer gaming at high frequencies exhibit increased gray matter volume in the hippocampus (15). Moreover, patients with IGD show heightened bilateral hippocampal activation in response to gaming-related cues presented via social media. Notably, both the severity and duration of IGD are positively associated with activation levels in the left hippocampus, implicating its role in cue reactivity and the disorder’s progression (16). Altered volume and FC of the hippocampus and amygdala in individuals with IGD may be associated with aberrantly enhanced memory processes for gaming-related cues (17). In IGD patients, regional homogeneity (ReHo) values in the left hippocampus and right amygdala are negatively correlated with the P3 amplitude of event-related potentials, suggesting impaired integration of cognitive and sensory processing (18). These findings underscore the critical involvement of amygdalo-hippocampal complex (AHC) dysfunction in the pathogenesis of IGD. However, prior research has primarily focused on localized structural and functional alterations within the AHC. To date, no study has systematically examined the spatiotemporal functional dynamics of the AHC within large-scale brain networks.

Neural activity in the brain exhibits inherent temporal dynamics (19). Most existing fMRI studies on IGD assume signal stability during scanning, overlooking the evolving nature of brain networks over time. Dynamic functional network connectivity (dFNC), utilizing sliding window and clustering techniques, enables the detection of temporal fluctuations in network connectivity and reveals integrative brain mechanisms that static analyses may overlook (20). Previous studies have demonstrated that patients with substance use disorders exhibit dynamic reconfiguration of brain network connectivity (21). However, to date, no studies have investigated dynamic changes in brain networks among adolescents with IGD.

This study is the first to utilize dFNC analysis to examine alterations in the AHC among adolescents with IGD, and to investigate the association between abnormal AHC connectivity and cognitive decline in IGD patients. We hypothesize that adolescents with IGD exhibit aberrant dFNC between the AHC and other brain networks, and that these altered dFNC patterns are associated with cognitive impairments in IGD adolescents. This study aims to provide novel evidence for the involvement of the AHC in the pathophysiology of IGD.

## 2 Methods and materials

### 2.1 Participants

The IGD group consisted of adolescents recruited from the outpatient and inpatient psychiatric departments of the First Affiliated Hospital of Zhengzhou University between September 2022 and June 2025. Inclusion criteria for the IGD group: (1) aged 12–18 years, no gender restriction; (2) Asian ethnicity, right-handed; (3) meeting DSM-5 diagnostic criteria for Internet Gaming Disorder as diagnosed by psychiatrists.

The HC group consisted of adolescents recruited from the community between September 2022 and June 2025. The recruitment process involved posting study posters in public venues such as local community centers, youth activity centers, and libraries, as well as publishing recruitment advertisements through community social media groups. Inclusion criteria: (1) aged 12–18 years, no gender restriction; (2) Asian ethnicity, right-handed.

Common exclusion criteria for all groups: (1) history of severe traumatic brain injury or organic brain disorders (e.g., encephalitis, epilepsy); (2) intellectual disability; (3) current or previous diagnosis of psychiatric disorders (e.g., depressive disorders, anxiety disorders, obsessive-compulsive disorder, bipolar disorder, schizophrenia); (4) family history of mental illness or genetic diseases; (5) history of drug abuse or substance abuse; (6) contraindications for MRI examination. Additional exclusion criteria for the IGD group: use of psychiatric medications within the past month, or receiving other forms of treatment such as physical therapy or psychotherapy.

During the recruitment period, a total of 96 adolescents met the diagnostic criteria for IGD. Among them, 16 voluntarily withdrew from the study, 3 did not complete the scale assessments in full, 5 presented with comorbid depressive episodes, and 1 was diagnosed with comorbid obsessive-compulsive disorder. Consequently, a final total of 71 adolescents with IGD were included in the study. A total of 63 HC adolescents agreed to participate in the study. Of these, 1 did not complete the scale assessment and 1 was diagnosed with a depressive episode. Therefore, a final total of 61 HC adolescents were included in the study. The study recruited 132 participants for functional MRI scanning. Following quality control procedures, 9 participants were excluded due to excessive head motion (>3 mm displacement), resulting in a final sample of 123 participants.

### 2.2 Psychological scale assessments

All participants completed the Young’s Internet Addiction Scale (YIAS) (22). The scale consists of 20 items using a 5-point scoring system, with a total score of 100 points. It is categorized into: mild (40 ≤ YIAS score < 60), moderate (60 ≤ YIAS score < 80), and severe (YIAS score ≥ 80) levels. The higher the total score, the more severe the degree of internet addiction in adolescents with IGD.

Using the MATRICS Consensus Cognitive Battery (MCCB) to assess the cognitive functions in 7 dimensions (Speed of Processing, Attention/Vigilance, Working Memory, Verbal Learning, Visual Learning, Reasoning and Problem Solving, Social Cognition) (23). The MCCB is increasingly used for the assessment of neurocognitive function in psychiatric disorders (24, 25).

### 2.3 MRI data collection

This study used a 3.0T Magnetom Prisma MRI scanner from Siemens, Germany. Before scanning, participants were instructed to lie supine with eyes open, breathe calmly, and avoid intentional cognitive activity. The scanning sequence and parameters are as follows: rs-fMRI scan: The sequence is the Blood-oxygen-level-dependent (BOLD) sequence, with parameters of TR 1000ms, TE 30ms, flip angle 70°, slice thickness 2.2mm, number of slices 52, matrix 64×64, voxel size 3mm×3mm×3mm, and scanning time 360s.

### 2.4 fMRI data preprocessing

Data preprocessing was performed using the DPABI toolbox on the MATLAB platform, with the following steps: (1) Data format conversion; (2) Removal of initial time points: the first 10 time points were discarded; (3) Slice timing correction: using the middle slice as reference to eliminate interleaved acquisition timing differences; (4) Head motion correction: subjects with head motion exceeding 3mm displacement or 3° rotation were excluded; (5) Spatial normalization: structural images were first co-registered to functional space, then segmented into gray matter, white matter and cerebrospinal fluid while generating transformation matrices. A study-specific template was created using all participants’ data, to which all images were registered before normalization to Montreal Neurological Institute (MNI) space (resampled voxel size: 3mm×3mm×3mm); (6) Spatial smoothing: performed using a 6mm full-width-at-half-maximum Gaussian kernel; (7) Nuisance covariate regression: regressing out head motion parameters, cerebrospinal fluid, white matter, and global brain signals.

### 2.5 Group independent component analysis

The group independent component analysis (gICA) was performed using the GIFT software (http://icatb.sourceforge.net/). This is a commonly used method in fMRI data analysis, which can decompose fMRI signals into multiple independent components (IC). Compared to other brain networks, the area of AHC is smaller (26, 27). To obtain a more detailed IC, this study employed a relatively higher number of ICs (200). The 200 ICs were decomposed and independent spatial maps were generated. Previous literature, visual inspection and the spatial correlation values between ICs and the template were used for IC selection. Effective network components were selected from 200 ICs by Stanford University’s functional ROI template. The correlation values greater than 0.2 were considered as valid ICs. The spatial maps of each individual subject’s selected intrinsic connectivity network (ICN) were converted into Z values. The Z value of each spatial map represents the contribution intensity of its time course to the ICs.

### 2.6 Dynamic FNC analyses

To observe fluctuations in FC during resting state, a sliding window approach was employed to calculate dFNC between IC time series. The procedure included the following steps: (1) Extraction of time series from regions of interest; (2) Setting window parameters: time series were segmented into multiple windows using a window size of W = 22 TRs and step size of T = 1 TR; (3) Time series segmentation: division of time series into consecutive windows according to specified parameters, with each window containing multiple time points; (4) Computation of windowed FC matrices: for each window, connectivity matrices were generated by calculating similarity measures between time series. The median FNC value within the window was used to represent the dFNC strength.

### 2.7 Statistical analysis

Statistical analyses were performed using two-sample t-tests and chi-square tests to compare demographic and clinical characteristics. For all clinical characteristics data analyses, we used IBM SPSS Statistics 22.0. Statistical significance was set at P < 0.05. Using Spearman correlation analysis, the correlations between the dFNC intensities of AHC and other ICs and the MCCB T values in each state were compared. A false discovery rate (FDR) adjustment was made for the multiple comparisons.

To investigate the potentially mediating effects of dFNC of AHC on mediating the relationship between the severity of addiction to IGD and cognitive performance, a mediation analysis using the PROCESS macro was conducted (28). The significance of the mediation effect was assessed via a bootstrapping method, which involved generating 5,000 bias-corrected indirect effect estimates to construct 95% confidence intervals. This resampling technique is widely employed in statistical inference to estimate standard errors, establish confidence bounds, and evaluate model stability. A mediation effect was considered statistically significant if the 95% confidence interval did not include zero. For detailed analysis, please refer to the supplementary materials (**Supplementary Figure S1**).

### 2.8 Ethics

This study was approved by the Ethics Committee of the First Affiliated Hospital of Zhengzhou University (Approval No.2022-KY-0438-002) and complied with the Declaration of Helsinki. Before participating in this study, all participants and their legal guardians provided informed consent with signatures. During the psychological assessment, the evaluation process commenced only after both the adolescent participants and their family members had signed the informed consent forms. Family members were present throughout the entire assessment procedure.

## 3 Results

### 3.1 Demographics and scales

A total of 123 participants were included in this study, with 66 in the IGD group and 57 in the healthy controls (HC) group. There were no significant differences between the two groups in terms of age, gender, or years of education. Compared to the HC group, the IGD group had significantly higher YIAS scores (*P* < 0.001) and showed marked reductions in the T-scores of the MCCB and all subscale scores (*P* < 0.01). Detailed results are presented in **Table 1**.

**Table 1:** Clinical characteristics of all subjects.

Variables	IGD (n=66)	HC (n=57)	*P* value
Age (y)^b^	14.34 ± 1.80	13.82 ± 2.11	0.208
Gender(M:F)^a^	56: 10	48: 9	0.736
Education(y)^b^	9.24 ± 1.89	8.70 ± 2.10	0.206
Disease duration (y) ^b^	3.35 ± 0.97	NA	0.703
YIASb	62.83 ± 9.95	20.79 ± 1.58	<0.001
MCCB T-scoreb	36.77 ± 7.24	48.73 ± 8.13	<0.001
Speed of processing^b^	39.81 ± 9.69	49.08 ± 9.38	<0.001
Attention/vigilance^b^	33.98 ± 6.60	47.08 ± 11.10	<0.001
Working memory^b^	37.33 ± 6.66	51.08 ± 6.32	0.001
Verbal learning^b^	36.62 ± 6.48	48.68 ± 8.39	<0.001
Visual learning^b^	34.71 ± 11.33	47.05 ± 10.38	<0.001
Reasoning and problem solving^b^	37.59 ± 6.90	49.14 ± 6.38	<0.001
Social cognition^b^	37.36 ± 7.03	48.75 ± 6.35	0.003

a Chi square test, btwo-sample t-test.

IGD, internet gaming disorder; HC, healthy controls; NA, not applicable; M, male; F, female; y, year; YIAS, Young’s Internet Addiction Scale; MCCB, MATRICS Consensus Cognitive Battery.

### 3.2 Spatial distribution of ICNs

**Figure 1:**
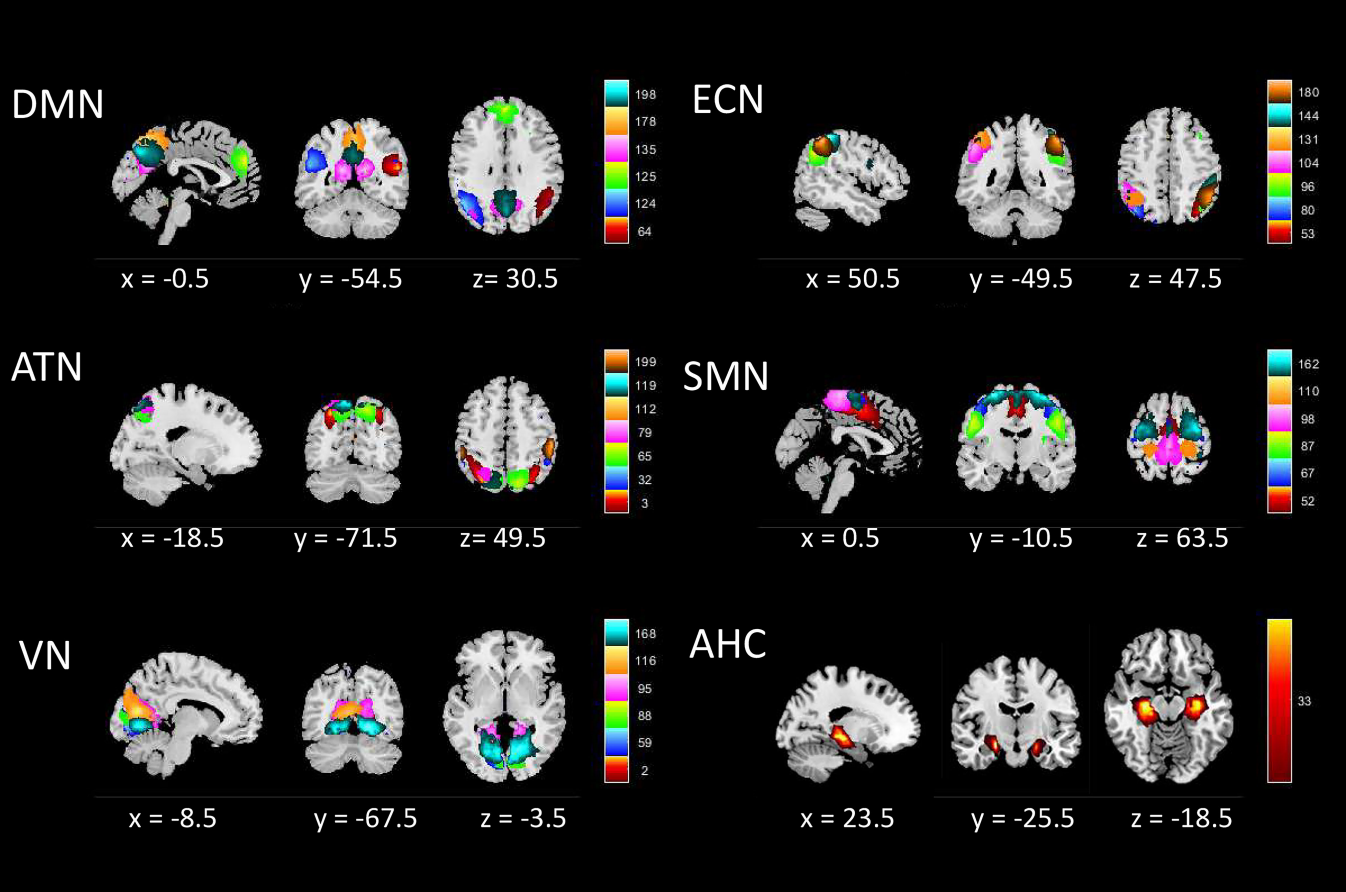
The spatial distrib ution map of independent components. DMN, Default mode network; ECN, Executive control network; ATN, Attention network; SMN, Sensorimotor; VN, Visual network; AHC, Amygdalohippocampal complex.

Using the GIFT method, 200 ICs were identified, and 33 ICs were assigned into six brain networks, whose spatial distributions are illustrated in **Figure 1**. These six networks include: the Default mode network (DMN) (IC64, IC124, IC125, IC135, IC178, IC198), the Executive control network (ECN) (IC53, IC80, IC96, IC104, IC131, IC144, IC180), the Attention network (ATN) (IC3, IC32, IC65, IC79, IC112, IC119, IC199), the Sensorimotor network (SMN) (IC52, IC67, IC87, IC98, IC110, IC162), the Visual network (VN) (IC2, IC59, IC88, IC95, IC116, IC168), and the Amygdala-Hippocampal Complex (AHC) (IC33). **Supplementary Figure S2A** depicts the mean FC across all subjects’ static ICs, while **Supplementary Figure S2B** presents the mean FC matrix illustrating the interactions among the six identified brain networks.

### 3.3 dFNC states

**Figure 2:**
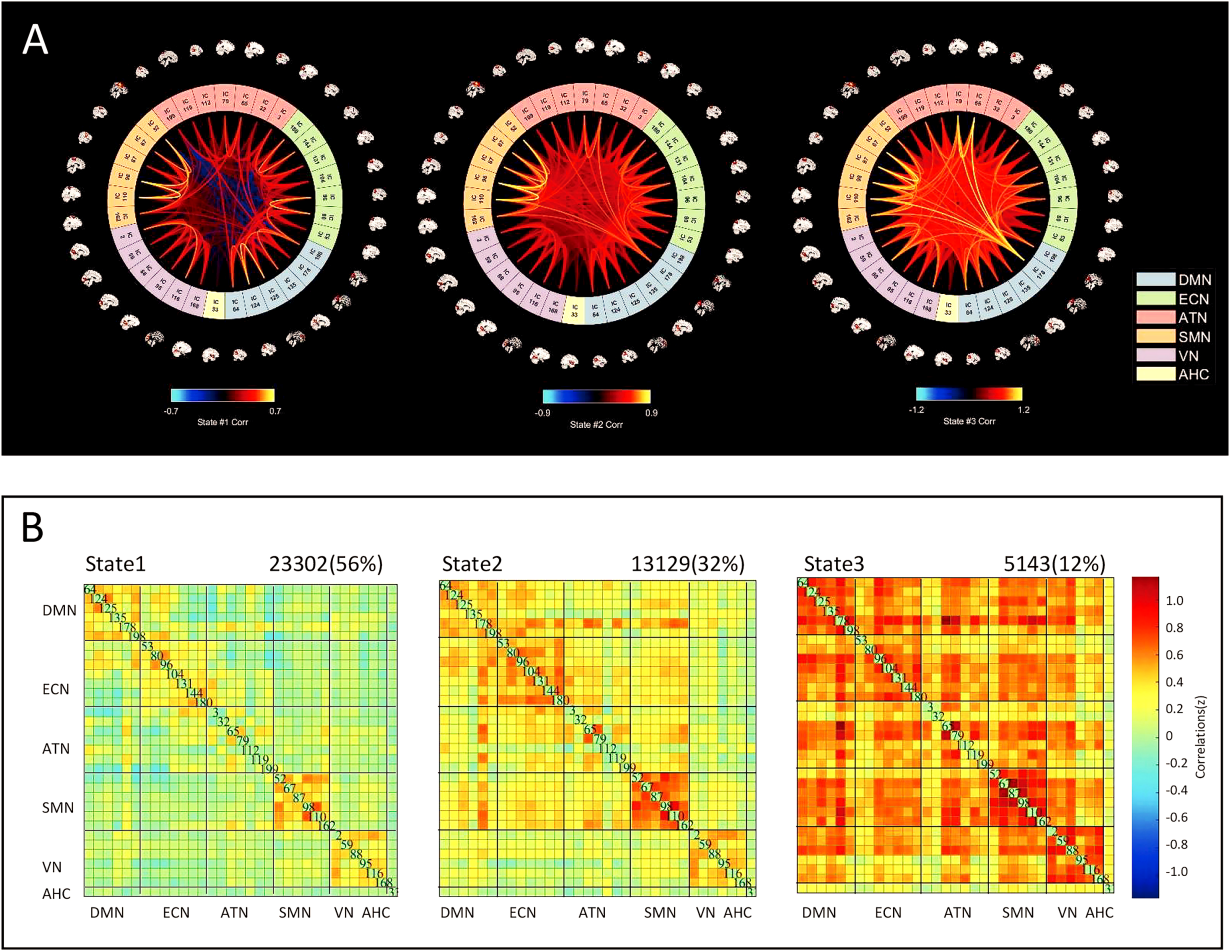
Cluster analysis results. **(A)** The functional connections in each state; **(B)** The cluster centroids of the 4 states. The topmost part shows the percentage of this state in the total number of all functional connection matrices. IC, independent component; DMN, Default mode network; ECN, Executive control network; ATN, Attention network; SMN, Sensorimotor; VN. Visual network; AHC, Amygdalohippocampal complex.

In this study, k-means clustering was used to identify three distinct FC matrix states. These states differed in their spatial FC patterns (**Figure 2**) and in the proportion of time each state occurred. The total occurrence percentages of these states varied, with State 1 being the most frequent (56%), followed by State 2 (32%) and State 3 (12%). State 1 was characterized by generally sparse connectivity. State 2 featured enhanced connectivity within the ECN, SMN, and VN, as well as increased connectivity between the DMN-SMN, DMN-ATN, and DMN-ECN. State 3 was characterized by widespread global connectivity enhancement across brain networks.

### 3.4 Correlation analysis of AHC’s dFNC and cognitive function

**Figure 3:**
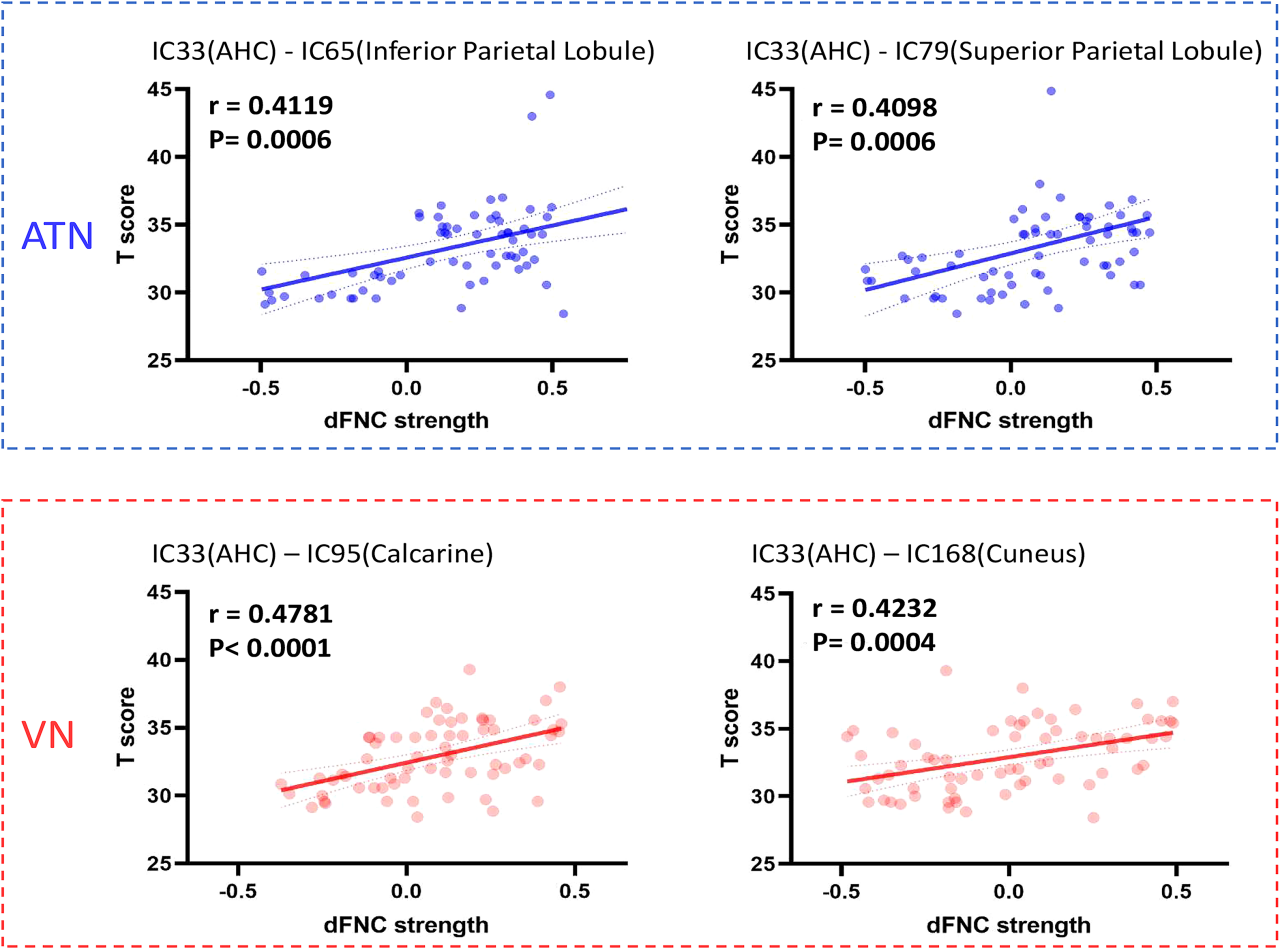
Correlation analysis results. IC, independent component; ATN, Attention network; VN, Visual network; dFNC, dynamic function network connectivity; AHC, Amygdalohippocampal complex.

We also assessed the relationship between changes in the dFNC of the AHC with other ICs and cognitive function in each state. The median FNC value within the window was used to represent the dFNC strength. As shown in **Figure 3**, in State 2, the dFNC strength between AHC (IC33)-ATN and AHC-VN was significantly correlated with the T-scores of the MCCB. Specifically, the involved ICs included: IC65: Inferior parietal lobule (*r* = 0.4119, *P* = 0.0006) and IC79: Superior parietal lobule (*r* = 0.4098, *P* = 0.0006) from the ATN, as well as IC95: Calcarine (*r* = 0.4781, *P* < 0.0001) and IC168: Cuneus (*r* = 0.4232, *P* = 0.0004) from the VN.

### 3.5 Mediation analysis

**Figure 4:**
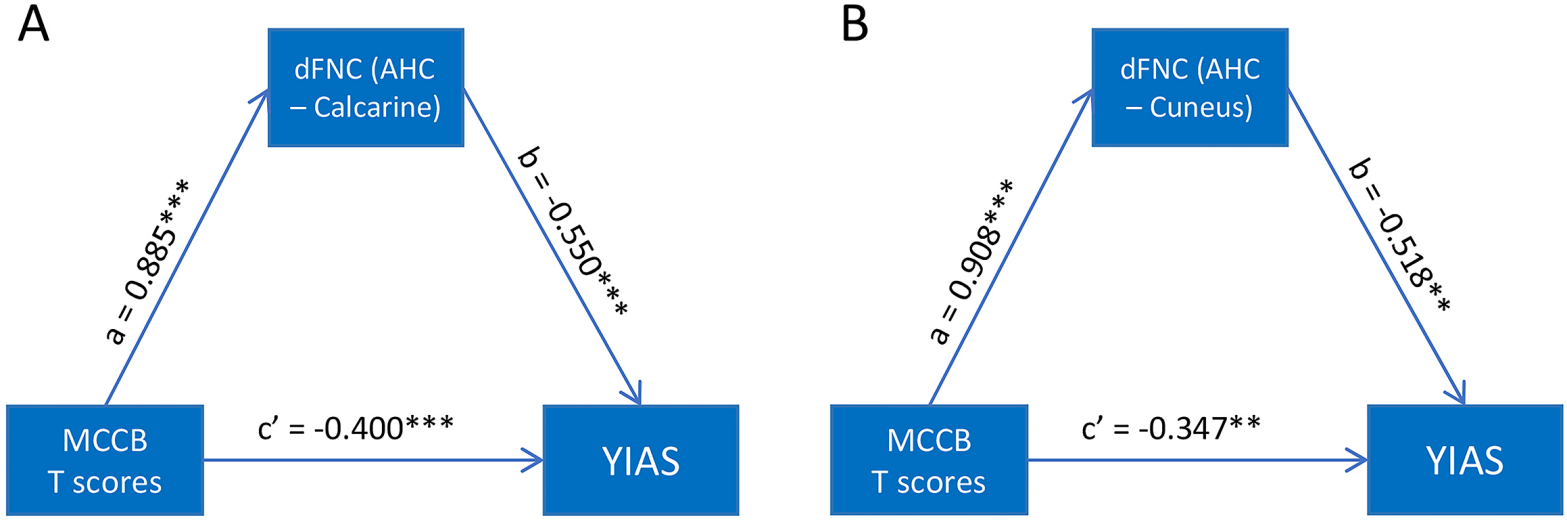
Mediation analyses results. **(A)** Mediation analysis of MCCB T scores (X) and YIAS scores (Y), with the dFNC(AHC-Calcarine) serving as the mediating variable (M); **(B)** Mediation analysis of MCCB T scores (X) and YIAS scores (Y), with the dFNC(AHC-Cuneus) serving as the mediating variable (M). MCCB, MATRICS Consensus Cognitive Battery; YIAS, Young’s Internet Addiction Scale; dFNC, Dynamic function network connectivity; AHC, Amygdalohippocampal complex. ***P < 0.001. **P < 0.01.

To further elucidate the relationship among MCCB scores, altered dFNC, and YIAS scores, we conducted a mediation analysis. The results revealed that, in state 2, dFNC alterations involving the AHC mediated the association between cognitive performance and the degree of internet addiction among adolescents with IGD. Specifically, dFNC between the AHC and the calcarine cortex, as well as between the AHC and the cuneus, exhibited significant mediation effects (**Figure 4**). The corresponding regression coefficients are presented in **Tables 2**, **3**.

**Table 2:** Mediation effect outcomes.

Coefficients	β	SE	t	P	LLCI	ULCI
Outcome: dFNC (AHC – Calcarine)						
(constant)	-1.985	0.142	-14.012	<0.001	-2.268	-1.702
MCCB T (a)	0.066	0.004	15.244	<0.001	0.057	0.074
Outcome: YIAS						
(constant)	124.571	16.519	7.54	<0.001	91.561	157.582
dFNC (AHC – Calcarine) (b)	-38.383	7.228	-5.311	<0.001	-52.826	-23.939
MCCB T (c’)	-2.068	0.534	-3.870	<0.001	-3.135	-1.000
Indirect effect (a*b)	-2.512	0.569	NA	NA	-3.454	-1.214

dFNC, Dynamic function network connectivity; AHC: Amygdalohippocampal complex; LLCI, lower level of the 95% confidence interval; NA, not applicable; SE, standard error; ULCI, upper level of the 95% confidence interval.

**Table 3:** Mediation effect outcomes.

Coefficients	β	SE	t	P	LLCI	ULCI
Outcome: dFNC (AHC – Cuneus)						
(constant)	-3.045	0.182	-16.703	<0.001	-3.409	-2.680
MCCB T (a)	0.096	0.005	17.368	<0.001	0.085	0.107
Outcome: YIAS						
(constant)	140.158	24.313	5.765	<0.001	91.572	188.745
dFNC (AHC – Cuneus) (b)	-2.456	0.761	-3.228	0.002	-3.977	-0.936
MCCB T (c’)	-15.588	7.202	-2.164	0.003	-29.980	-1.195
Indirect effect (a*b)	-1.496	1.005	NA	NA	-2.849	-1.023

dFNC, Dynamic function network connectivity; AHC, Amygdalohippocampal complex; LLCI, lower level of the 95% confidence interval; NA, not applicable; SE, standard error; ULCI, upper level of the 95% confidence interval.

## 4 Discussion

This study is the first to investigate large-scale brain network alterations in dFNC involving the AHC in adolescents with IGD. Our findings yield three key insights. First, clustering analysis identified three distinct brain states, each characterized by unique connectivity patterns. Second, dFNC alterations between the AHC and both the ATN and the VN were significantly associated with cognitive performance in IGD group. Third, aberrant AHC-VN dFNC served as a mediator linking cognitive impairment to internet addiction severity. Collectively, these findings suggest that dynamic AHC connectivity may serve as a potential neural marker of early cognitive dysfunction in adolescent IGD, offering new insights into its neuropathological mechanisms.

### 4.1 Neurophysiological basis of the AHC

The AHC, comprising the amygdala and the hippocampal formation, plays a pivotal role in cognitive functioning, particularly in emotional regulation, memory processing, and cognitive control (29, 30). These two structures are functionally interconnected (27, 31). The amygdala modulates emotional responses, which can in turn enhance hippocampus-dependent memory encoding and consolidation, a process that is both robust (32) and enduring (33). Conversely, the hippocampus also contributes to emotional processing, particularly in relation to negative affective states such as disappointment and dysphoria (34), and can modulate affective responses through memory retrieval mechanisms (35). This bidirectional interaction underscores the critical role of the AHC in emotional memory and learning (36). Neuroimaging studies have demonstrated that the AHC is strongly involved in craving responses to addiction-related cues (37). Exposure to drug-associated stimuli has been associated with increased dopaminergic transmission in the AHC (38). In individuals with IGD, decreased gray matter density in the hippocampus and reduced white matter integrity in the amygdala have been reported (39). In contrast, some studies have reported increased volumes in these regions, with hippocampal volume showing a positive correlation with IGD symptom severity (17). Cognitive dysfunction in IGD has also been associated with impaired hippocampal and amygdalar function, reflecting the cumulative effects of habitual gaming and altered emotional memory encoding (18).

This study is the first to identify the amygdalo-hippocampal IC in adolescents with IGD, emphasizing its potential as a sensitive neural biomarker for early cognitive changes. This novel discovery highlights the critical functional role of the AHC in adolescent IGD and establishes a foundation for future research exploring its dynamic connectivity patterns during the preclinical phase of cognitive deterioration.

### 4.2 The dynamic connection of AHC

In the cluster analysis, we identified three recurring states over time, each exhibiting a distinct dFNC pattern, reflecting the brain’s flexibility in coordinating functional activity among networks. The FC between networks reflects the capacity for functional integration across different brain regions (40), where sparse connectivity often indicates inefficient functional integration (41). State 1 was characterized by globally sparse connectivity, with no prominent increases or decreases in FC. This pattern may reflect an early stage of the disorder, during which connectivity reductions begin to emerge but have not yet become widespread or irreversible. In State 2, superimposed on a globally sparse connectivity pattern, increased intra-network connectivity was observed within the ECN, SMN, and VN, along with enhanced inter-network connectivity between the DMN and SMN, ATN, and ECN. This pattern may reflect a compensatory mechanism. The combination of globally reduced yet locally enhanced connectivity suggests a reallocation of neural resources during the progression of IGD, potentially aimed at preserving or enhancing cognitive function (42, 43). State 3 was characterized by a globally elevated level of connectivity, with highly synchronized interactions across multiple networks compared to State 2. Such widespread connectivity enhancement, particularly between the DMN and ECN, has been associated with compensatory mechanisms during the progression of brain disorders (44, 45), potentially serving to preserve cognitive function. The increased synchronization observed in State 3 likely represents a neurobiological mechanism underlying cognitive resilience, enabling enhanced integration of emotional and executive processes, functions essential for complex behaviors including decision-making, attention, and emotional regulation.

Correlation analysis revealed that, in State 2, the dFNC strength between the AHC and both the ATN and VN was significantly associated with MCCB T-scores. This finding suggests that cognitive impairment in adolescents with IGD is linked to disrupted AHC-related network connectivity, consistent with previous reports (18). However, in contrast to earlier studies that primarily focused on localized structural and functional abnormalities in the amygdala and hippocampus, our results emphasize the importance of large-scale dFNC between the AHC and both ATN and VN in the pathophysiology of IGD. Aberrant connectivity between the AHC and these networks may reflect difficulties in emotional and memory regulation in adolescents with IGD, potentially leading to alterations in visual association, attention, interpretation, and regulatory processes—ultimately manifesting as cognitive decline (46).

### 4.3 Mediating role of the AHC

Mediation analysis revealed that reduced AHC-related dFNC mediated the relationship between poorer cognitive performance and the degree of internet addiction among adolescents with IGD. Specifically, the calcarine cortex and cuneus, key regions within the VN, emerged as critical mediators. Weaker dFNC between the AHC and these VN regions was associated with more severe internet addiction symptoms. Given the VN’s role in processing and interpreting visual information, its functional integrity is closely tied to cognitive function in individuals with IGD (47). Cognitive ability is known to be inversely associated with internet addiction severity, and reduced AHC–VN connectivity may further exacerbate addictive behaviors. Previous studies have shown that individuals with high gaming engagement exhibit significantly stronger FC between the DAN and VN compared to those with low engagement, suggesting that abnormal DAN–VN interactions contribute to IGD development (48). Adolescents with IGD often spend prolonged periods fixated on computer screens during gaming, leading to sustained visual stimulation that may impair visual attention and processing over time (49). Such impairments are typically detectable in corresponding brain regions (50). The cuneus, a critical component of the VN (51), has been associated with personality traits such as harm avoidance, which correlates with its gray matter density (52). Additionally, sensation-seeking behaviors have been linked to cuneus activation in response to novel stimuli (53). These findings suggest that, as cognitive function declines, altered connectivity between the AHC and VN regions, particularly the calcarine cortex and cuneus, may mediate the worsening of IGD symptoms in adolescents. Taken together, this study advances our understanding of the tripartite relationship among the degree of internet addiction among adolescents with IGD, cognitive performance, and dynamic FC.

This study has several limitations. First, as a case–control design, it cannot establish causal relationships between IGD and brain network alterations in adolescents. Longitudinal studies are needed to clarify these potential causal links. Second, the sample included a higher proportion of male participants, likely due to the higher prevalence of IGD among males. Future studies should explore sex-related differences in resting-state brain network dynamics among adolescents with IGD. Third, the relatively small sample size may limit the generalizability of the findings. Larger-scale studies are warranted to further investigate dFNC alterations in adolescent IGD using more robust datasets.

## 5 Conclusion

These findings suggest that aberrant dynamic FC of the AHC, particularly its disrupted interactions with the VN, may represent a core neural mechanism underlying cognitive impairments in adolescents with IGD. This study provides novel evidence for the neurobiological basis of IGD and highlights the utility of dynamic network analysis in uncovering the pathophysiology of behavioral addictions.

## Data Availability

The original contributions presented in the study are included in the article/**Supplementary Material**. Further inquiries can be directed to the corresponding author.
