# Supplementary material for: Dynamic functional connectivity of the amygdala-hippocampal complex is associated with cognitive impairment in adolescents with Internet gaming disorder
Source: Front Psychiatry. 2025 Nov 26;16:1689119. doi: 10.3389/fpsyt.2025.1689119 (PMC12689908; doi:10.3389/fpsyt.2025.1689119)
Supplement: Supplementary file 1 [file Supplementaryfile1.docx]

**Supplementary materials**

**Methods:**

**Methods S1: Mediation analysis**

This analysis assessed the overall effect of variable X on Y (c), which includes the indirect effect of X on Y through mediator M (a × b) and the direct effect of X on Y (c’). Significance tests employed 5000 bootstrap samples, with a significant indirect effect defined as a 95% confidence interval (CI) that excluded zero. Statistical significance was set at p < 0.05. See Figure S1.

**
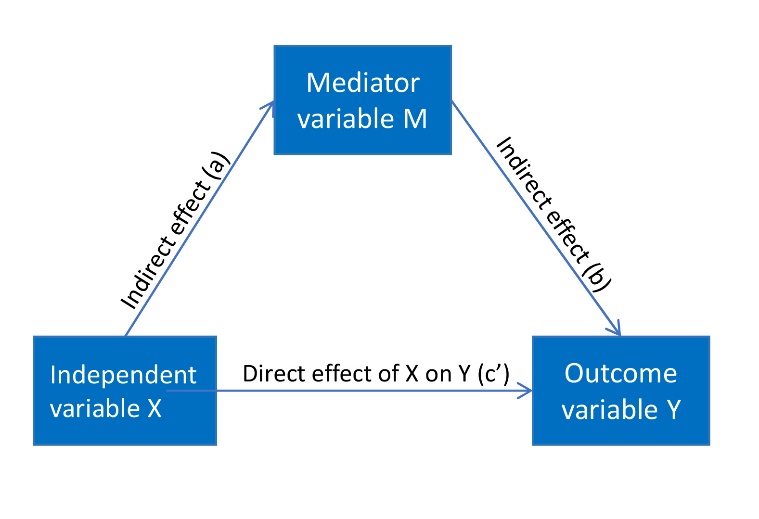
**

Figure S1. Schematic representation of mediation analysis.

**Results:**
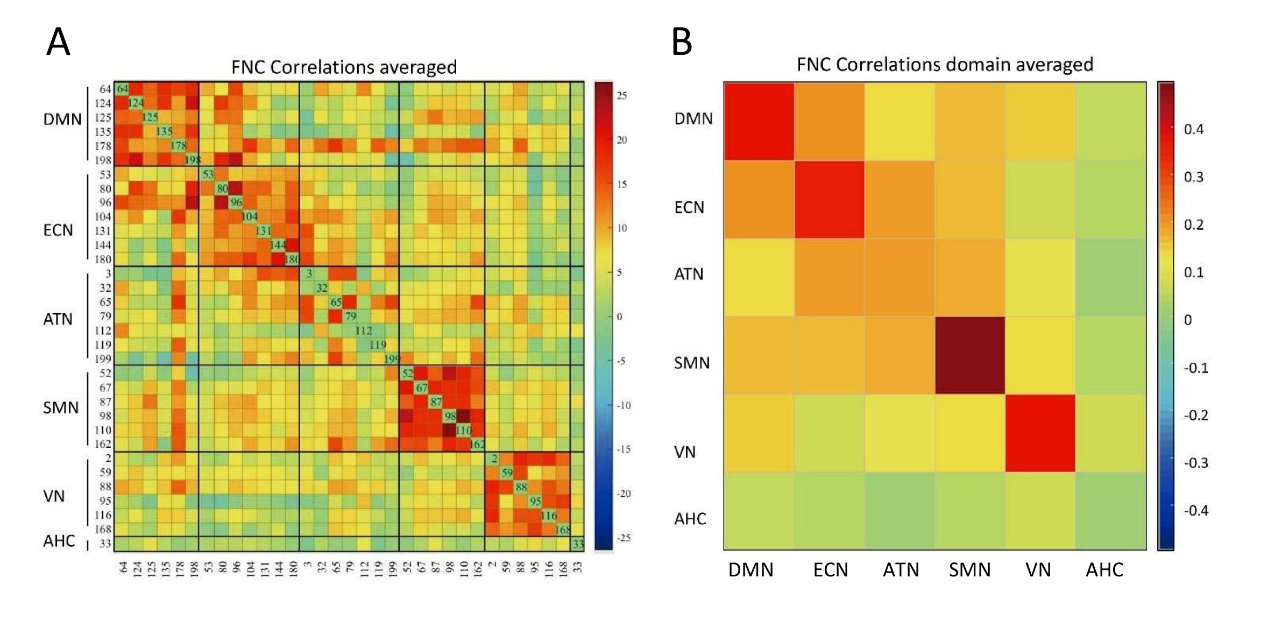


Figure S2. Average FC at the group level. (A) The group-level average FC between independent components was visualized as a matrix graph. (B) The average FC between static brain networks at the group level for all subjects was displayed in the matrix graph. Abbreviations: FC: functional connectivity; FNC: functional network connectivity; DMN: Default mode network; ECN: Executive control network; ATN: Attention network; SMN: Sensorimotor; VN: Visual network; AHC: Amygdalohippocampal complex.
